# Some Observations and Experiments Connected with Oral Electricity

**Published:** 1878-05

**Authors:** Henry S. Chase

**Affiliations:** St. Louis, Mo.


### ARTICLE II.

### *Some Observations and Experiments Connected with Oral Electricity.*

BY HENRY S. CHASE, D. D. S., M. D., ST. LOUIS, MO.

For the sake of simplicity I will speak of electrical action and galvanic action as one. Their slight differences need not be discussed. The electric current is always the same, however produced. To construct a battery, two substances must be used having different capacities for resisting decomposition. These two may be in close contact, or they may be separated and connected by another substance which will conduct a current of electricity.

If I tie together a piece of gold and a piece of tin, and put the same into weak nitric acid which will dissolve tin, I have a galvanic battery. The tin is acted upon and dissolved; the gold is not acted upon at all, and the tin is dissolved *more* rapidly, and will lose *more* weight in a given time than it would if not united to the gold. The gold, then, may be said to *hasten* the destruction of the tin. If I unite the tin to *silver* instead of gold, the tin will not lose so much in the same given time as though united to gold. Now, although the tin and the silver are placed in the same strength of acid as that of the tin and the gold, the silver will lose nothing when united to the tin, but if placed in the same acid by itself, it would lose more or less in weight. The silver here is saved at the expense of the tin.

Now I will take a copper wire (gold, silver, or other metal will answer,) two feet long, and fasten one end of the wire to the gold plate, and the other end to the tin plate, and put the two plates into the same acid, keeping the plates half an inch apart. I now cut the copper wire and attach the two cut ends to a galvanometer. The needle of the galvanometer *instantly* shows a deflection of, say 80 degrees. If I lift the *plates* out of the acid, the needle instantly returns to zero. This shows that an electrical current is instantly produced when the acid touches the *plates*. Now, I will use a silver plate instead of a gold one, with the tin plate, the needle instantly flies *towards* 80 degrees, but does not reach it, because the electrical current is not as powerful as that produced by gold and tin. Experiments prove that the most powerful current is produced by two different substances that are the farthest apart on the *electro chemical* scale. Let us take either gold, or platinum, or carbon, for one plate (or element) of the battery, because neither of these will be dissolved by a simple acid. Now we will take zinc or tin for the other plate (or element,) and now we have elements or plates which *are* far apart on *the scale*.

In making a scale of electrical power (potential scale) we place at the head those substances that are the most easily dissolved in acids. Let us make one from substances used as filling materials: Ox. chlo. zinc; Hill's stopping; tin; amalgams; gold. No current can be produced in the battery excepting at the *expense* of one of the *elements*. The stronger the current the more rapidly must one of the plates (elements) dissolve. Between ox. chl. zinc and gold, the current, therefore, would be the most powerful. Currents produced by the union of any two of the other elements would be far less powerful than between ox. chl. zinc and gold, if the conductivity of the zinc was better than it really is. The *weakest* current would take place between ox. chl. zinc and Hill's stopping. Those elements which stand *close together* on the potential scale produce the *weakest* current. Consequently, If Hill's stopping and ox. chl. zinc be used as plates (elements) the weakest of all currents would be evolved. Between tin and amalgams there would be a weak current.

Now I will add another element to the potential scale which must imperatively have a place in oral electricity, and that is dentos or tooth substance. Between dental amalgams and gold there is a strong current, because they are far apart on the potential scale; although there is nothing that we use as a filling material standing *between* amalgams and gold.

Now, we must put next to the *top* of the potential scale DENTOS. Then it will read thus: Ox. chl. zinc, dentos, Hill's stopping, tin, amalgams, gold.

The two plates of a battery are called *elements.* That one which is the most easily dissolved in an acid is called the *positive* element. If the elements are tin and gold, tin is the positive element, and gold the negative one. Now, if tin and dentos are the elements, then dentos will be the positive one, and tin the negative. Tin, then, is negative to all above it on the potential scale, and positive to all below it. And the same is true of any and all of the other elements named in the scale. According to the principles of electrical science, dentos is positive to all substances except ox. chl. zinc, used for filling decayed teeth.

If tin suffers by being in contact with gold in the presence of an acid, the dentos would suffer much more, as it is higher on the potential scale. Hundreds of experiments prove that dentos *is* next to the top of the scale. The position of elements on the scale is determined by dissolving in acids. Experiments prove that dentos in contact with gold, in the presence of an acid, does lose more in weight in a given time than dentos does in contact with amalgams, tin, ox. chl. zinc, or Hill's stopping.

I have made many experiments with dentos and filling material, as follows: Ten (10) cubes of elephant's tusk (dentine) of equal density, size and weight, each having a cavity of the same size by drilling through the cube, were soaked in water one week, and then weighed. Each cube had its weight marked on its surface in *centigrams* (1·6 gr.) Two cubes were filled with gold foil; two were filled with tin and silver amalgam; two were filled with tin foil; two were filled with Hill's stopping; two were filled with ox·chl. zinc. These were placed in vinegar and water, and remained one week. At the end of that time the plugs were removed and the cubes alone weighed. Each cube had lost in weight as follows:

| | | |
|---|---|---|
| Ox. chl. zinc, cube lost - | 00 | centigramme. |
| Hill's stopping, " " - - | 1 | " |
| Tin, " " - - | 2 | " |
| Amalgam, " " - - | 3 | " |
| Gold, " " - | 5 | " |

At another time the loss in weakened lemon juice was as follows:

| | | |
|---|---|---|
| Ox. chl. zinc, cube lost - | 1 | centigramme. |
| Hill's stopping, " " - - | 2 | " |
| Tin, " " - | 3 | " |
| Amalgam, " " - - | 4 | " |
| Gold, " " - | 6 | " |

Many similar experiments were tried with varying results, according to the size of the cubes and the acids employed, but in all cases the cubes containing gold lost a greater percentage than those containing other material. These experiments may be valuable in pointing out the relative safety of different substances united to *living* dentos, but they cannot be considered absolute. Living dentos may have *some* power to protect itself from galvanic action, but I think it must be slight in the case where galvanic action only takes place (in a *water-tight* plug,) just at the *margin* of the fillings, where it would be least likely to receive nutrition. Where a plug admits saliva between itself and dentos, galvanic action takes place at every point of contact between the plug and the former whenever the admitted fluid becomes acidulous. In much of the dentos here, the power of self-protection may be something more than theoretical. At a certain point of acidity it must succumb to the attack-force, as may be seen by knowing that decay or chemical dissolution of dentos is continually taking place in the mouth. Whatever power of self-protection sound and living dentos has, its weakness in this respect is that which has created the dental profession.

The following experiment may be interesting: Take two polished plates of soft steel. precisely alike, each having three holes exactly the same size. The holes in one plate are plugged with gold foil ; the holes in the other plate are plugged with tin foil. Place them in a dish of river water; the latter barely wets the top surface of the plates. At the end of one hour the plate having *gold* in it will be *covered with rust* on its top surface ; the plate with *tin* will not be at all rusted.

The conductivity of our filling materials have the following order: Gold conducts electricity the best ; tin next ; amalgam ; Hill's stopping. I place gold at 60 ; tin, 30 ; amalgams, 10 ; Hill's stopping, 3. The conductivity of these substances has been *determined by experiment.*

Conductivity is an important factor in the galvanic action of dental plugs. This is why a water-tight amalgam plug is better than a water-tight tin foil plug *in acid conditions of the mouth ;* the latter being a better conductor.

I deduce from these experiments that every plugged tooth is a complete galvanic battery ready for action when the necessary stimulant (acids) is applied.

That Hill's stopping will arrest decay a long time better than gold in certain conditions of the mouth. That ox. chl. zinc will arrest decay *perfectly* for a very *short time*, as it is itself destroyed too rapidly.

That tin foil is superior to gold for arresting decay a *limited* time in acid conditions determined by its progress in cupping on the surface.

That amalgams which do not *leak* arrest decay better than gold or tin in acid conditions.

That gold is the *worst* arrestor of decay in acid conditions, whether with a tight or leaky plug—though far worse if leaky.

That a water tight gold plug arrests decay as well as any other material when the contents of the mouth are *not acidulous.*

That a water-tight plug of one material is as good as another for arresting decay in a *neutral* condition of the oral fluids, so long as its *form* is not changed by natural attrition or accident.

That in acid conditions a filling material which is a *bad conductor* of electricity will arrest decay better than one that is a superior conductor.

That for this reason a gutta percha filling arrests decay in a remarkable manner, and that when a water-tight plug is made with it, there is nothing superior as long as it retains its integrity of mass.—*Dental Quarterly.*

